# Supplementary material for: Influence of Parental Health Locus of Control on Behavior, Self-Management and Metabolic Control, in Pediatric Patients with Type 1 Diabetes
Source: J Pers Med. 2022 Sep 27;12(10):1590. doi: 10.3390/jpm12101590 (PMC9604908; doi:10.3390/jpm12101590)
Supplement: Supplementary file 1 [file jpm-12-01590-s001.zip › jpm-1887984-supplementary.pdf]

Supplementary Material S1: Topic guides of the semi-structured interview. The research methodology involves individual or paired narrative interviews conducted in a semi-structured manner following the macro-themes (topics) presented below. The topic guide is a flexible interview guide.

Topic 1: Illness representation
- Personal beliefs related to illness
- Influence of moral and/or religious orientations on the representation of illness

Topic 2: Learning
- Caregiver/patient education
- Peer learning (caregiver-caregiver; patient/patient)

Topic 3: Management
- Daily disease management practices
- Division of family tasks in illness management

Topic 4: Illness in everyday life contexts
School
Home
Leisure time

Supplementary Material S2: Linear regression analysis for coefficient variation of mean HbA1c in the last year as a dependent variable and demographic, auxologic, metabolic, family and caregiver data as independent variables, reporting only statistically significant results.

**Independent variables**	**All patients**	
	**P**	**R2**
**Age at the study enrollment (years)**	**0.013**	0.045
**HbA1c at the study enrolment (%)**	**<0.0001**	0.830
**Total daily insulin (UI/Kg/die)**	**<0.0001**	0.160
**% of time with active sensor**	**<0.0001**	0.161
**% of time in range (70-180 mg/dL)**	**<0.0001**	0.388
**Mean glucose (mg/dL)**	**<0.0001**	0.491
**Coefficient of variation (CV) (%)**	**0.006**	0.064
**Physical activity (hours/week)**	**0.004**	0.056
**Parent education level**	**0.004**	0.062
**Working status**	**0.002**	0.070

Supplementary Material S3: Correlations between internal and external HLOC scores. The categories 'Parent' and 'Child' (1 and 6) refer to an internal HLOC, whereas health professionals, the media, God and fate (2; 3; 4; 5) identify an external HLOC.

All patients

	**Internal HLOC score**	**External HLOC score**
**Age (y)**	p 0.59	p 0.06
**Mean HbA1c in the last year (%)**	**p 0.02**, r -0.20	p 0.37
**% of time in range (70-180 mg/dL)**	**p 0.009**, r 0.23	**p 0.012**, r -0.22

**Coefficient of variation (CV) (%)**	p 0.24	**p 0.039**, r 0.18
**Parent's education level**	p 0.71	**p <0.0001**, r -0.35
**Parent's working status**	p 0.70	**p <0.0001**, r -0.46
**Nationality**	p 0.75	**p 0.006**, r 0.25
**Religion**	p 0.95	**p <0.0001**, r 0.32

HbA1c >7% group

	**Internal HLOC score**	**External HLOC score**
**Age (y)**	p 0.55	**p 0.015**, r -0.27
**Parent's education level**	p 0.70	**p 0.013**, r -0.28
**Parent's working status**	p 0.75	**p 0.002**, r -0.34
**Religion**	p 0.58	**p 0.024**, r 0.26

HbA1c ≤7% group

	**Internal HLOC score**	**External HLOC score**
**Sex**	p 0.41	**p 0.011**, r -0.33
**Pubertal stage**	**p 0.03**, r 0.29	p 0.96
**Age at T1D onset (years)**	p 0.28	**p 0.038**, r 0.28
**Diabetes duration (years)**	p 0.94	**p 0.026**, r -0.30
**BMI z-score**	**p 0.027**, r 0.29	p 0.17
**School education level**	**p 0.012**, r 0.33	p 0.52
**Parent's education level**	p 0.55	**p 0.010**, r -0.34
**Parent's working status**	p 0.83	**p <0.0001**, r -0.47
**Nationality**	p 0.45	**p 0.002**, r 0.42
**Religion**	p 0.65	**p 0.001**, r 0.44
